# Clinical metabolomics reveals potential diagnostic biomarkers in serum samples from patients with generalized ligamentous laxity

**DOI:** 10.3389/fmolb.2025.1554936

**Published:** 2025-05-30

**Authors:** Yu Zhang, Xiaochao Hu, Feng Chen, Tongtong Liu, Ping Cai, Shijia Liu, Luning Sun

**Affiliations:** ^1^ Affiliated Hospital of Nanjing University of Chinese Medicine, Jiangsu Province Hospital of Chinese Medicine, Nanjing, China; ^2^ School of Pharmacy, Nanjing University of Chinese Medicine, Nanjing, China; ^3^ Pharmaceutical Sciences Research Center, Department of Pharmacy, Children’s Hospital of Nanjing Medical University, Nanjing, China

**Keywords:** generalized ligamentous laxity, anti-inflammatory, metabolomics, osteoarthritis, biomarkers

## Abstract

**Objectives:**

Discovering the potential metabolic alterations underlying generalized ligamentous laxity (GLL) is crucial for identifying new therapeutic targets and improving patient prognosis. Serum metabolites could mirror systemic and local alterations and help understand the metabolic features of GLL. The present work aimed to determine serum biomarkers for GLL diagnosis and to unveil metabolic pathways linked to GLL.

**Design:**

Prospective, observational cohort study.

**Methods:**

In this study, serum sample collection was conducted from 65 GLL and 35 healthy control (HC) cases. The obtained specimens were assessed by ultra-performance liquid chromatography high-resolution mass spectrometry (UPLC-HRMS). Orthogonal partial least squares-discriminant analysis (OPLS-DA), random forest (RF), binary logistic regression (BLR) and receiver operating characteristic (ROC) analyses were applied to screen and validate biomarkers.

**Results:**

Totally 24 small-molecules were considered differentially expressed metabolites. Of these, hexadecanamide was found to be a specific biomarker for differential diagnosis of GLL, with an area under the ROC curve (AUC) of 0.907. Additionally, the α-linolenic acid and linoleic acid metabolism had the most substantial alteration among various pathways in GLL cases. The altered pathway of α-linolenic acid and linoleic acid metabolism affected bone mineral density and bone metabolism in GLL patients, leading to enhanced inflammation or fracture of the bone and joints. Joint inflammation and dislocation led to systemic ligament relaxation, which induced and aggravated musculoskeletal injury.

**Conclusion:**

Through identification of serum biomarkers and analysis of metabolic pathways, the current study provided novel insights into GLL pathogenesis.

## 1 Introduction

Generalized ligamentous laxity (GLL) is an orthopedic condition characterized by soft tissue injury, ligamentous laxity, and excessive joint mobility, leading to symptoms such as neck and back pain, lumbar disc herniation, and reduced quality of life (Cho et al., 2019; Cahill et al., 2020; Tobias et al., 2013). The prevalence of GLL ranges from 10% to 30% in the general population (Cahill et al., 2020). Due to the abnormal development of ligament structures, GLL patients are at higher risk of joint injuries, osteoarthritis, and even visceral prolapse (Patterson et al., 2013; Liu et al., 2011). Early detection of GLL is crucial for effective prevention and management.

The Beighton Score (BS) is the most commonly used tool for diagnosing GLL, with a score of four or higher indicating a positive result (Malek et al., 2021). However, the BS has limitations in accurately reflecting GLL, as it focuses primarily on upper limb joints and omits many major joints, leading to potential misclassification and inadequate detection of hypermobility (Malpas et al., 2018). This highlights the need for more reliable diagnostic biomarkers to improve GLL diagnosis and treatment.

Biomarkers are essential for detecting disease occurrence and progression by reflecting changes in biological structures and functions (Keeratichamroen et al., 2020). Metabolomics, a high-throughput “-omics” technique, has been successfully applied to identify clinical biomarkers for various diseases by elucidating metabolic pathways and providing insights into disease mechanisms (Malpas et al., 2018). Serum biomarkers are valuable for their non-invasive nature, accessibility, stability, and clinical relevance. They reflect systemic metabolism and pathological changes, making them essential for diagnosis, monitoring, and prognosis (Lazaros et al., 2025). Easily obtained during routine check-ups, serum samples cause minimal discomfort and remain stable for long-term storage and analysis. Regular blood tests enable dynamic monitoring of disease progression and treatment response, especially in chronic and critical illnesses (Lee et al., 2024; Zhang et al., 2025; Thorlacius-Ussing et al., 2023). Extensive clinical literature supports their use across various diseases, providing a strong foundation for our study.

LC-MS, integrating the separation efficiency of liquid chromatography with the high-sensitivity detection of mass spectrometry, enables the simultaneous detection of hundreds to thousands of low-abundance metabolites in serum. For instance, in Alzheimer’s disease research, HPLC-MS/MS achieved accurate quantification of 337 ceramides, identifying 62 differential molecules as potential biomarkers (Ge et al., 2025). In vaccine research, the sensitivity of UPLC-MS/MS reached 0.5 ng/mL, allowing for the detection of highly polar metabolites (Shinde et al., 2025). LC-MS requires minimal sample volumes, making it suitable for trace samples like serum. A single serum sample can be used across multiple platforms, such as NMR and LC-MS, with optimized sample preparation (e.g., acetonitrile precipitation, MWCO filtration) to accommodate various metabolite classes (Kacerova et al., 2025). Coupled with bioinformatics tools, LC-MS supports high-throughput data generation and analysis. In a study on hoof deformation in dairy cows, integrating LC-MS metabolomics with ICP-OES ionomics revealed regulatory mechanisms of the metabolite-ion network (Deng et al., 2023a). In ceramide analysis, mathematical models improved the accuracy of qualitative identification through retention time prediction and fragment pattern analysis (Ge et al., 2025). LC-MS has shown remarkable performance in biomarker discovery and mechanistic studies across various diseases. In non-alcoholic steatohepatitis (NASH), sCDCP1 identified by LC-MS achieved an AUROC of 0.838 for risk stratification (Vali et al., 2023). In placenta accreta spectrum (PAS) research, LC-MS discovered that L-arginine promotes cell invasion via the GPRC6A/PI3K/AKT pathway, offering a therapeutic target (Gao et al., 2025).

**FIGURE 1:**
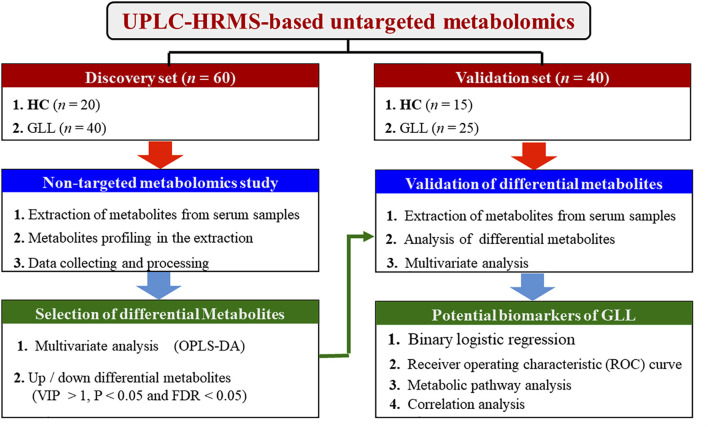
Study workflow.

In this study, we aim to identify new biomarkers for GLL diagnosis and management through untargeted metabolomics of serum samples from GLL patients and healthy controls (HCs), as depicted in Figure 1.

## 2 Materials and methods

### 2.1 Patients

Between October 2018 and August 2021, a total of 35 HCs and 65 GLL cases were enrolled in the Affiliated Hospital of Nanjing University of Chinese Medicine. The current study had approval from the Institutional Review Board (IRB) of the Affiliated Hospital of Nanjing University of Chinese Medicine and complied with the declaration of Helsinki. The approval ethical file number was No. 2018NL-056-02. All patients with GLL were clinically evaluated with the BS system. A score of four or greater was used to determine the presence of GLL. Table 1 summarizes the clinicodemographic features of the participants.

**TABLE 1:** Clinicodemographic characteristics of the study participants.

Characteristic	Discovery set		Validation set	
	GLL	HC	GLL	HC
Number	40	20	25	15
Male/Female	18/22	9/11	11/14	7/8
Age, mean [min, max]	36.58 [12, 59]	38.60 [30, 50]	34.83 [15, 44]	35.40 [22, 49]
Beighton score [min, max]	7.50 [5, 9]	-	7.71 [6, 9]	-

Exclusion criteria were: (1) complications with other GLL disorders; (2) complications with severe primary diseases such as those affecting the liver, kidney, cardiovascular, cerebrovascular and hematopoietic systems; (3) mental illness with inability to cooperate; (4) involvement in clinical studies within the last 1 month; and (5) unwillingness to participate in the research. The collected serum specimens were immediately placed at −80°C for future assessment.

### 2.2 Sample preparation and metabolomics profiling

The chromatography was performed using an ExionLCTM high-performance liquid chromatography system (America AB SCIEX company). In positive ion mode, a Waters HSS T3 column (100 × 2.1 mm, 1.7 µm) was used to analyze small polar metabolites. The column temperature was maintained at 40 °C, and the sample plate temperature was kept at 4 °C. The sample volume and flow rate were set at 2 μL and 0.3 mL/min, respectively. Mobile phase A was ultra-pure water containing 0.1% formic acid (FA), and mobile phase B was 100% acetonitrile. In negative ion mode, an Acquity UPLC BEH Amide column (100 × 2.1 mm, 1.7 µm) was used for large polar metabolites. Mobile phase A was ultra-pure water containing 5 mM NH4OAc and 0.05% FA, and mobile phase B was 100% acetonitrile. Gradient elution was employed in both modes.Mass spectral data were acquired using a TripleTOFTM 5600+ high-resolution mass spectrometer (America AB SCIEX company). First-order spectra were obtained by full scanning, and second-order spectra were acquired by information-dependent acquisition (IDA).

In electrospray ionization-mass spectrometry (ESI-MS), metabolites often appeared as multiple ion species due to isotopologues, adducts, clusters, and in-source fragments. These species shared the same retention time as the parent compound. XCMS algorithm detected features with signal intensity exceeding a threshold at specific m/z values. However, some features could be attributed to instrumental noise or artifacts. One compound might have multiple features due to isotopic peaks or adducts, complicating statistical analysis and compound identification. To further verify and accurately annotate metabolites, we used MS2 data from standard samples. By comparing the MS2 fragmentation patterns of experimental samples with those of standard samples, we could more accurately confirm the structures of metabolites. The MS2 data of standard samples provided us with characteristic fragmentation patterns of known metabolites under specific conditions, which played a key role in our study, helping us distinguish and identify the structures of different metabolites.

### 2.3 Data analysis

The MSConvert software was used for format conversion of the original data. The WIFF format of raw data was not recognized and should be converted to the XLS format. The primary mass spectrum peak table with all material mass-to-charge ratio information and secondary data quality control were imported into XCMS, MetDNA and Masterview Software. Suitable experimental conditions were selected and matched in the database for qualitative analysis, and the above software programs were utilized to identify various compounds based on retention time, accurate mass number, secondary mass spectrometry, etc. Supplementary Material provides the specific process for data analysis using the above three software packages. The MultiQuant software was used for the quantitative peak areas of qualitative compounds.

### 2.4 Metabolomics analysis

The data were standardized using MetaboAnalyst v5.0. The standardized data were analyzed by orthogonal partial least squares discriminant analysis (OPLS-DA) to identify differentially expressed metabolites (DEMs) between GLL cases and healthy controls (HCs). DEMs were selected based on variable importance in projection (VIP) values (VIP >1.0), Mann–Whitney-Wilcoxon test p values (p < 0.05), false discovery rate (FDR) values (FDR <0.05), and fold change (FC > 1.1 or <0.6).Random forest (RF) and binary logistic regression (BLR) models were used to determine the best combination of DEMs. Receiver operating characteristic (ROC) curves were generated, and areas under the ROC curves (AUCs) were assessed to evaluate the potential of these metabolites as GLL biomarkers. Metabolite pathway analysis was performed using the KEGG database, and the associations between potential biomarkers and the clinical index (BS) were analyzed by Spearman correlation.Data analysis for ROC and BLR was performed using SPSS 23.0.

## 3 Results

### 3.1 Participants’ features and study design

Totally 60 participants (40 GLL cases and 20 HCs) were allocated to the test set for evaluating biomarkers, and 40 (25 GLL cases and 15 HCs) to the validation set for further evaluation of potential biomarkers. Although previous results from epidemiological analyses showed that GLL could develop at any age, GLL was more frequent in females than in male patients (Ortiz-Declet et al., 2022)]. Therefore, the clinical features (Table 1) corroborated the distribution characteristics and age and sex data of GLL patients in clinic. The BS values of the GLL and HC groups showed a significant difference. Due to the relatively good range of activity and physical condition of patients with mild GLL, symptoms related to mild GLL are often ignored by patients. In clinical practice, it is difficult to encounter mild GLL patients with a BS value of 4. In this study, the BS values of the 65 recruited GLL cases were all greater than 4.

### 3.2 Metabolomics of serum specimens from GLL and HC cases

**FIGURE 2:**
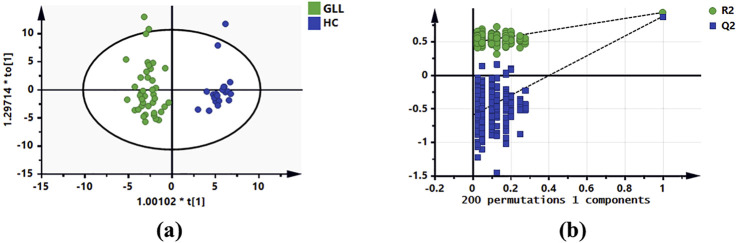
OPLS-DA of serum metabolomics data for GLL patients and HCs. **(a)** OPLS-DA score plot based on the HCs and GLL groups in the test set. **(b)** Validation plots constructed from 200 random permutation tests showing the robustness of the original OPLS-DA model.

In the metabolomics analysis, 88 small-molecule metabolites were identified in serum specimens in the test set. The selected metabolites were submitted to OPLS-DA, in which GLL cases and HCs were clearly separated (Figure 2a). The results of the permutation test revealed the OPLS-DA model showed substantial robustness in reflecting metabolic differences between the GLL group and HCs, with no significant overfitting (Figure 2b). Therefore, the collected clinical samples were able to cover the relevant needs of this study. Subsequently, 38 significantly altered metabolites were identified in the test set (VIP>1, *p* < 0.05, FDR<0.05 and FC > 1.1 (or <0.6). The important information and statistical analysis results of 38 DEMs are listed in Supplementary Table S1, S2, respectively. Principal component analysis (PCA) was performed cluster the control samples and GLL samples (Supplementary Figure S2). The quality control samples are tightly clustered and intermingled with the study samples, indicating good consistency and controllability of the data throughout the experimental process and suggesting the absence of significant batch effects or other sources of variation that might affect the reliability of the results.

### 3.3 Identification and performance of potential diagnostic biomarkers

In this study, the selection process was divided in three steps: (1) DEMs underwent sorting in descending order based on VIP and *p* value; (2) DEMs should show consistent trends in the test and validation datasets; and (3) select metabolites further underwent screening by RF model, BLR and ROC analyses.

**FIGURE 3:**
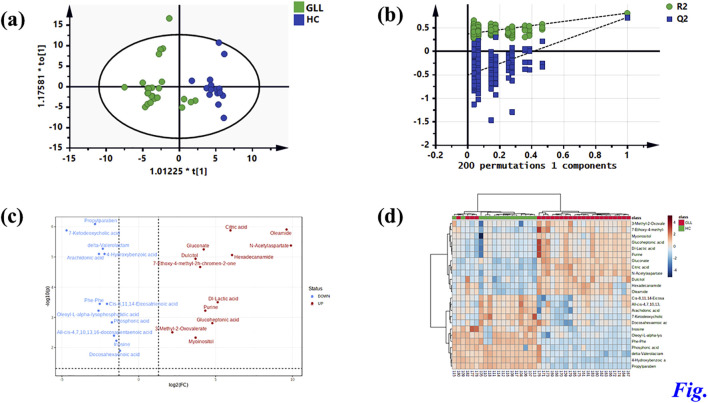
Identification of potential metabolic biomarkers for GLL diagnosis in the validation set. **(a)** OPLS-DA score plot based on the HCs and GLL groups in the validation set. **(b)** Validation plots constructed from 200 random permutation tests showing the robustness of the original OPLS-DA model. **(c)** Volcano plot of 24 differential metabolites. **(d)** Heatmap of 24 differential metabolites.

Untargeted metabolomics detected 38 DEMs between the GLL group and HCs in the test set. Serum specimens from the validation set were utilized for further evaluation of the 38 DEMs for reliability, screening major metabolites as possible diagnostic biomarkers for GLL. OPLS-DA of GLL cases versus HCs showed an overt between-group separation (Figure 3a) in the validation set, corroborating OPLS-DA findings in the test dataset. Additionally, permutation testing confirmed the OPLS-DA model was reliable in the prediction of variations between GLL cases and HCs in the validation set (Figure 3b). The above 38 DEMs were further examined in the validation set as described for the test set. Interestingly, 24 of 38 metabolites had significant differences between GLL cases and HCs, with consistent trends in the test and validation datasets (Table 2). To highlight the significant differences in metabolites in the validation set, volcano plots were utilized to depict the 24 DEMs (Figure 3c), with red and blue dots each representing 12 upregulated (*p* < 0.05 and FC > 1.1) and downregulated (*p* < 0.05 and FC < 0.6) metabolites, respectively. Furthermore, for visualizing the distributions of DEMs in various groups in the validation dataset, a hierarchical clustering algorithm (HCA) was utilized to carry out cluster analysis of the identified DEMs (Figure 3d). In conclusion, 12 of 24 DEMs, namely, citric acid, oleamide, gluconate, N-acetyl aspartate, dulcitol, hexadecanamide, seven-ethoxy-4-methyl-2H-chromen-2-one, Dl-lactic acid, purine, glucoheptonic acid, 3-methyl-2-oxovalerate and myoinositol, were elevated, while the remaining 12 DEMs were decreased in patients with GLL relative to HCs.

**TABLE 2:** Differentially altered metabolites between GLL patients and HCs.

Metabolite	Test set				Validation set			
	VIP^a^	P value^b^	FDR^c^	FC^d^	VIP	P Value	FDR	FC
3-Methyl-2-oxovalerate	2.09	<0.001	<0.001	27.3↑	1.17	0.003	0.007	2.04↑
4-Hydroxybenzoic acid	1.41	<0.001	<0.001	0.44↓	1.93	<0.001	<0.001	0.50↓
7-Ethoxy-4-methyl-2H-chromen-2-one	1.74	<0.001	<0.001	3.43↑	1.25	<0.001	<0.001	2.26↑
7-Ketodeoxycholic acid	1.20	<0.001	<0.001	0.12↓	1.74	<0.001	<0.001	0.17↓
All-cis-4,7,10,13,16-docosapentaenoic acid	1.59	<0.001	<0.001	0.35↓	1.13	0.004	0.009	0.46↓
Arachidonic acid	1.50	<0.001	<0.001	0.29↓	1.42	<0.001	<0.001	0.29↓
Cis-8,11,14-Eicosatrienoic acid	1.48	<0.001	<0.001	0.43↓	1.22	<0.001	<0.001	0.42↓
Citric acid	1.73	<0.001	<0.001	21.4↑	1.94	<0.001	<0.001	8.87↑
Delta-valerolactam	1.35	<0.001	<0.001	0.44↓	1.87	<0.001	<0.001	0.33↓
Dl-Lactic acid	1.27	<0.001	<0.001	2.13↑	1.32	<0.001	<0.001	2.07↑
Docosahexaenoic acid	1.45	<0.001	<0.001	0.41↓	1.10	0.013	0.026	0.58↓
Dulcitol	1.65	<0.001	<0.001	5.09↑	1.44	<0.001	<0.001	2.95↑
Glucoheptonic acid	1.20	<0.001	<0.001	2.09↑	1.24	0.001	0.004	2.01↑
Gluconate	1.48	<0.001	<0.001	6.40↑	1.75	<0.001	<0.001	3.72↑
Hexadecanamide	1.57	<0.001	<0.001	7.58↑	1.37	<0.001	<0.001	5.85↑
Inosine	1.06	<0.001	<0.001	1.14↑	1.18	0.006	0.013	1.12↑
Myoinositol	1.12	<0.001	<0.001	2.21↑	1.04	0.004	0.010	1.71↑
N-acetyl aspartate	1.57	<0.001	<0.001	16.1↑	1.90	<0.001	<0.001	16.06↑
Oleamide	1.63	<0.001	<0.001	7.55↑	1.58	<0.001	<0.001	6.79↑
Oleoyl-L-alpha-lysophosphatidic acid	1.19	<0.001	<0.001	0.47↓	1.26	<0.001	0.001	0.26↓
Phe-Phe	1.75	<0.001	<0.001	0.13↓	1.72	<0.001	<0.001	0.16↓
Phosphoric acid	1.31	<0.001	<0.001	0.42↓	1.51	0.001	0.003	0.49↓
Propylparaben	1.61	<0.001	<0.001	0.23↓	2.11	<0.001	<0.001	0.17↓
Purine	1.202	<0.001	<0.001	2.01↑	1.29	<0.001	0.001	2.05↑

^a^ The VIP, value was obtained from the OPLS-DA, model with a threshold of 1.0.

^b^ *P* values were obtained from one-way ANOVA.

^c^ The FDR, was obtained from the adjusted P value calculated using the MetaboAnalyst 5.0 software.

^d^ The FC, was obtained by comparing metabolites between GLL, patients and HCs.

**FIGURE 4:**
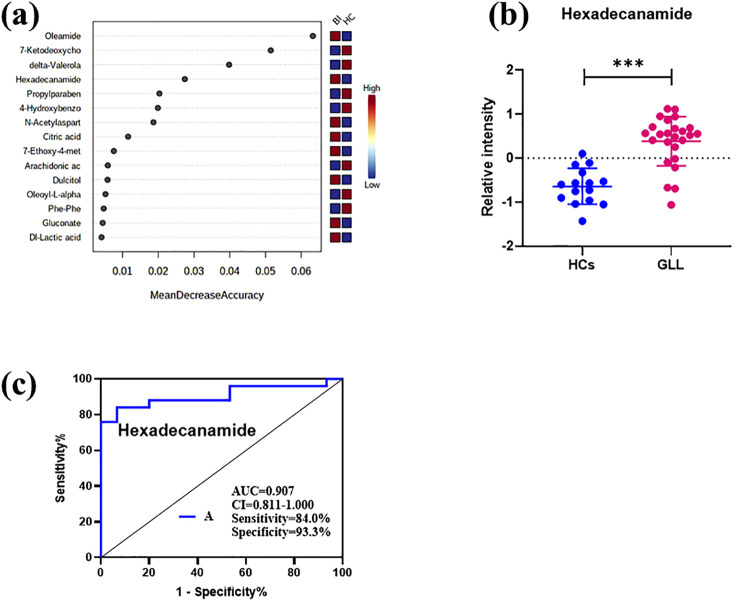
**(a)** Mean Decrease Accuracy values of the metabolic biomarkers used for random forest classification. **(b)** Scatter plot of the relative plasma strength of hexadecanamide. **(c)** ROC analysis of hexadecanamide.

**FIGURE 5:**
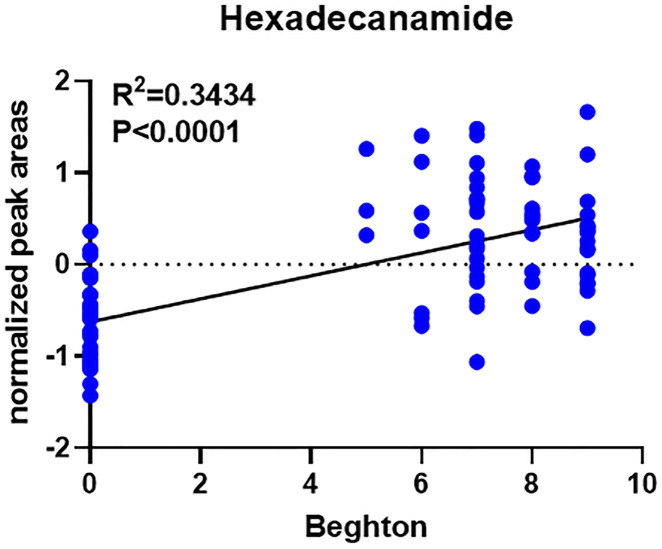
Correlation analysis between the biomarker hexadecanamide and the Beighton score.

In comparison with other methods, the RF model provides a more effective way to screen biomarkers in metabolomic analysis. Notably, 15 of 24 DEMs were selected using RF analysis (Figure 4a), and oleamide, 7-ketodeoxycholic acid, delta-valerolactam, hexadecanamide and propylparaben were the top 5 DEMs based on mean decrease accuracy (MDA). Subsequently, the above 15 DEMs were further examined by BLR in the validation set. Utilizing a forward stepwise optimization algorithm (Wald), hexadecanamide was detected as a reliable DEM in regression analysis. The representative chromatogram and secondary mass spectra of hexadecanamide are depicted in Supplementary Figure S1. Hexadecanamide’s relative intensity in serum is shown in Figure 4b. The commonest tool for assessing biomarkers for diagnostic accuracy is ROC curve analysis. As shown in Figure 4c, the AUC of hexadecanamide was 0.907, with a sensitivity of 84.0% and a specificity of 93.3% (95% confidence interval [CI] 0.811–1.000). ROC curve analysis confirmed the high potential of hexadecanamide to discriminate GLL cases from HCs. Finally, the biological significance of hexadecanamide as a potential biomarker in GLL was examined, and hexadecanamide had a positive correlation with the BS (Figure 5). The latter findings further suggested hexadecanamide could constitute a diagnostic biomarker in GLL. The diagnostic capability of this biomarker is provided in Supplementary Material.

### 3.4 Metabolic pathways

**FIGURE 6:**
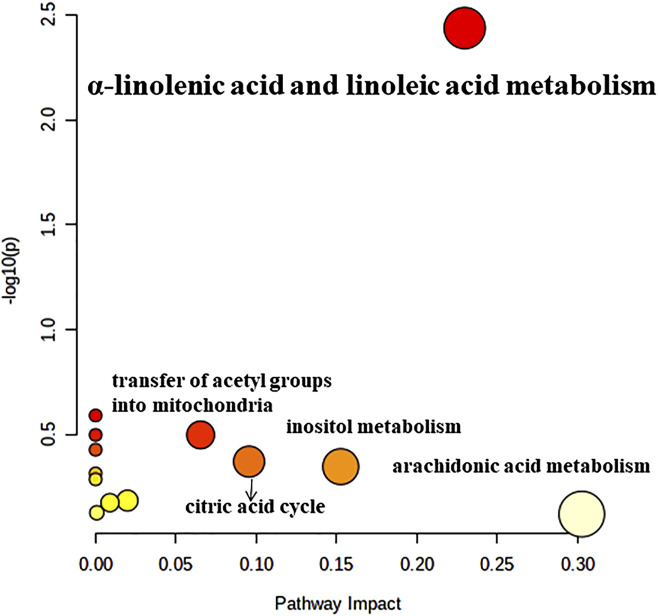
Pathway analysis of the differentially altered metabolites identified in patients with GLL.

To detect the biological significance of the above 24 DEMs, the KEGG database was utilized to identify pathways impacted by these metabolites. As shown in Figure 6, α-linolenic acid and linoleic acid pathways had significant alterations over the entire course of GLL and may be involved in GLL development. In addition, the detailed results of metabolic pathways are shown in Supplementary Material.

## 4 Discussion

In this study, UPLC-HRMS was applied to assess serum metabolites in GLL patients and HCs, and 15 metabolites were significantly different between these two groups as examined by the OPLS-DA and RF methods. A potential biomarker model was established to identify a biomarker (hexadecanamide) for diagnosing patients with GLL. Current diagnostic approaches for GLL predominantly rely on the Beighton Score, which assesses joint hypermobility through clinician-dependent subjective evaluations. Although this method is simple, rapid, and cost-effective, its outcomes are susceptible to inter-observer variability due to differences in clinical expertise, thereby limiting its sensitivity in identifying early-stage or mild GLL cases. Furthermore, it fails to capture molecular-level mechanisms underlying disease progression and carries a risk of underdiagnosis. In contrast, our proposed strategy employs liquid chromatography-mass spectrometry (LC-MS) to analyze serum metabolic profiles, could leverage quantitative metabolomic data to minimize human-induced biases. This approach also could enable the detection of molecular alterations prior to the manifestation of clinical symptoms while simultaneously uncovering dysregulated metabolic pathways. Notably, multivariable analysis highlighted marked differences in metabolomic profiles in GLL cases versus HCs, suggesting significant effects of disease state on these serum metabolites. As demonstrated above, 88 metabolites were screened, including 24 metabolites that were considered DEMs, including 12 each showing upregulation and downregulation (Table 2; Figure 3c). Among 24 DEMs, all-cis-4,7,10,13,16-docosapentaenoic, arachidonic and docosahexaenoic acids were involved in fatty acid metabolism, inflammation and immune regulation; 7-ketodeoxycholic acid, glucoheptonic acid, delta-valerolactam, Dl-lactic acid and dulcitol in the inflammatory response, pain and immune regulation; and cis-8,11,14-eicosatrienoic acid in fatty acid metabolism. These results indicated that majority of the identified DEMs play crucial roles in fatty acid metabolism, inflammation, pain, immune regulation in GLL. Hence, recent data provided strong support on the notion that inflammatory and metabolic disorders are the main culprit in GLL pathogenesis.

Analyzing untargeted metabolomic data, hexadecanamide was identified as a biomarker that could be utilized to diagnose GLL. Hexadecanamide is an endogenous fatty acid amide of the family of nuclear factor agonists. Hexadecanamide was shown to bind to nuclear receptors, affecting a variety of chronic pain- and inflammation-related biological functions (Roy et al., 2016). The primary target is thought to be peroxisome proliferator-activated receptor α (PPAR-α). Hence, hexadecanamide could be regarded as a serum biomarker of GLL. The results of this study indicated that hexadecanamide could segregate GLL cases from HCs. ROC curve analysis was conducted to assess hexadecanamide for diagnostic value in GLL, and an overt separation of GLL cases and HCs was achieved, with elevated diagnostic performance, sensitivity and specificity (Figure 4C). Hence, hexadecanamide as a diagnostic biomarker of GLL, would be conducive to increasing diagnostic accuracy, enabling early diagnosis, improved patient categorization, and monitoring of GLL treatment. This study innovatively provided a reference for the application of metabolomics in the clinical assessment of GLL and expanded research ideas on the pathogenesis of GLL. The present work was based on clinical practice and provided a reference for the translation of clinical results and the development of detection methods for GLL diagnosis.

The most significant pathways were α-linolenic acid and linoleic acid metabolism. The essential fatty acids (FAs) linoleic acid and α-linolenic acid are not synthesized in the human body, but are necessary for human health (Singh, 2005). In humans, arachidonic (ARA), eicosapentaenoic (EPA) and docosahexaenoic (DHA) acids are produced by these FAs, which play key roles in homeostatic modulation. Serum levels of α-linolenic acid and linoleic acid are positively correlated with BMD and inversely correlated with fracture risk (Lavado-García et al., 2018; Yuan et al., 2020). It was proven that a diet with a low *n*-6 (linoleic acid)/*n*-3 (α-linolenic acid) ratio, i.e., rich in *n*-3 polyunsaturated fatty acids (PUFAs), could protect bone and joint health (Saini and Keum, 2018; Albertazzi and Coupland, 2002). Previous studies have shown that adult males who initially present with muscle injury are more likely to develop GLL (Vasdev et al., 2016). Musculoskeletal injury may be an inducing factor of GLL and is closely related to ligamentous laxity (Ye et al., 2024). Therefore, it is speculated that changes in serum α-linolenic acid and linoleic acid metabolism in patients with GLL affect BMD and bone metabolism, leading to increased inflammation or fracture of the bone and joints (Deng et al., 2023b; Hoque et al., 2025). Joint inflammation and dislocation led to GLL, which induced and exacerbated musculoskeletal injury (Choi et al., 2024). The current work provided a solid basis for further research on GLL pathogenesis and treatment and screened potential biomarkers for the diagnosis of GLL (Talarico et al., 2024).

The current study had limitations. First, it had a limited sample size and involved a single center, and we expect both sensitivity and specificity to improve with additional samples from multiple centers. Additionally, metabolomics itself has inherent limitations. There is currently no single tool for detecting all metabolites in the same analysis, and the above data had incomplete metabolite annotation because multiple interesting pathways are not comprehensively explored. Besides, the data analysis approach might introduce biases in interpreting complex metabolic interactions. Future studies should prioritize larger cohorts, standardized metabolite validation protocols, and hybrid analytical methods combining machine learning with traditional statistics to enhance reliability. These improvements would strengthen the translational potential of findings.

## 5 Conclusion

Overall, nontargeted metabolomics was utilized for a comprehensive assessment of alterations of serum metabolites in GLL cases. We found alterations in multiple DEMs linked to critical biological processes, e.g., fatty acid metabolism, inflammatory response and immune regulation in GLL. Notably, we identified hexadecanamide as a potential diagnostic biomarker, which was further validated by RF, BLR and ROC analyses. In addition, α-linolenic acid and linoleic acid pathways had substantial perturbations over the entire course of GLL, affecting BMD and bone metabolism in patients, leading to more bone and joint inflammation or fractures. The above data provide novel insights into the improvement of GLL diagnosis and treatment, further enhancing the understanding of the pathophysiological mechanisms of GLL.

## Data Availability

The original contributions presented in the study are included in the article/Supplementary Material, further inquiries can be directed to the corresponding authors.
