# Supplementary material for: Clinical metabolomics reveals potential diagnostic biomarkers in serum samples from patients with generalized ligamentous laxity
Source: Front Mol Biosci. 2025 May 30;12:1554936. doi: 10.3389/fmolb.2025.1554936 (PMC12162281; doi:10.3389/fmolb.2025.1554936)
Supplement: Supplementary file 1 [file Supplementaryfile1.docx]

**1. Sample collection and preparation**

Fasting peripheral blood was collected from healthy controls (HCs) and glioblastoma multiforme (GLL) patients in the morning and allowed to coagulate at room temperature for 30-60 min. After centrifugation at 2000 rpm for 10 min, the upper serum layer was transferred to a sterile microcentrifuge tube. Each sample (100 μL serum) was mixed with 400 μL acetonitrile:methanol (1:1), vortexed for 30 s, sonicated in an ice bath for 10 min, and then stored at -20 ℃ for 1 h. Subsequently, the sample was centrifuged at 12000 rpm for 15 min at 4 ℃. The supernatant was dried and reconstituted with 100 μL acetonitrile:water (1:1), vortexed for 30 s, sonicated in an ice bath for 5 min, and centrifuged at 12000 rpm for 15 min at 4 ℃. A 10 μL aliquot of each sample was pooled as a quality control (QC) sample, while the remaining samples were stored separately. Two tubes (40 μL/tube) were prepared for column injection and stored at 4 ℃.

The sample collection and preparation followed the method described in XCMS, ensuring the stability and reproducibility of the analytical method. QC samples validated the stability and reproducibility of UPLC-HRMS, with most QC samples within twice the standard deviation (SD) in the score map and 98% of metabolomics variables showing a relative standard deviation (RSD) < 30%. PCA score plots in both ESI+ and ESI- modes demonstrated tight clustering of QC samples, indicating stable LC–MS system performance and high-quality data. The UPLC-HRMS method provided satisfactory stability and reproducibility for clinical metabolomics studies. The sample collection and preparation methods were stable and reliable, meeting the study's requirements.

**2. Metabolomics analysis using** **UPLC-HRMS**

The chromatography was performed using an ExionLCTM high-performance liquid chromatography system (America AB SCIEX company). In positive ion mode, a Waters HSS T3 column (100 × 2.1 mm, 1.7 µm) was used to analyze small polar metabolites. The column temperature was maintained at 40 ℃, and the sample plate temperature was kept at 4 ℃. The sample volume and flow rate were set at 2 μL and 0.3 mL/min, respectively. Mobile phase A was ultra-pure water containing 0.1% formic acid (FA), and mobile phase B was 100% acetonitrile. In negative ion mode, an Acquity UPLC BEH Amide column (100 × 2.1 mm, 1.7 µm) was used for large polar metabolites. Mobile phase A was ultra-pure water containing 5 mM NH4OAc and 0.05% FA, and mobile phase B was 100% acetonitrile. Gradient elution was employed in both modes.Mass spectral data were acquired using a TripleTOFTM 5600+ high-resolution mass spectrometer (America AB SCIEX company). First-order spectra were obtained by full scanning, and second-order spectra were acquired by information-dependent acquisition (IDA).

In electrospray ionization-mass spectrometry (ESI-MS), metabolites often appeared as multiple ion species due to isotopologues, adducts, clusters, and in-source fragments. These species shared the same retention time as the parent compound. XCMS algorithm detected features with signal intensity exceeding a threshold at specific m/z values. However, some features could be attributed to instrumental noise or artifacts. One compound might have multiple features due to isotopic peaks or adducts, complicating statistical analysis and compound identification.

**3. The specific process for data analysis using XCMS, MasterView and MetDNA software packages**

Data Preprocessing

Our study employed the freely available online XCMS for data preprocessing. This platform inherits the robust data preprocessing capabilities of XCMS, such as peak identification and alignment. Online XCMS supports various raw data formats, including netCDF, mzXML, mzData, and Agilent.d files. It also displays total ion chromatograms and retention time (RT) correction curves before and after RT correction to identify outliers.

Data Analysis Methods

Statistical Analysis:

Online XCMS provides univariate and multifactorial statistical methods, including paired t-tests and ANOVA for multiple comparisons.

SCIEX MasterView software, compatible with SCIEX QTRAP and TripleTOF systems, offers powerful MS2 data processing and spectral library search functions for accurate metabolite identification.

MetDNA software was used for metabolite annotation by matching MS1 peaks with MS2 spectra based on m/z (±25 ppm) and RT (±10 s) values. It employs a multi-step strategy to assign confidence levels to metabolite annotations, ranging from Grade 1 (annotated via standard spectral libraries) to Grade 4 (remaining unmatched peaks).

Machine Learning:

MetDNA removes annotation redundancy based on confidence levels, iteratively refining peak group composition until redundancy remains unchanged.

Parameter Settings

Statistical Thresholds:

p-value < 0.05.

Fold change (FC) > 1.1 or < 0.6.

Metabolite Annotation:

Confidence levels assigned based on spectral library matches, isotope peaks, and reliable adduct peaks.

Redundancy removal prioritizes higher confidence annotations.

Software Tools

Data Preprocessing: Online XCMS.

Statistical Analysis: Online XCMS, SCIEX MasterView.

Metabolite Annotation: MetDNA.

Visualization: Custom scripts or software interfaces for data visualization.

Results

A total of 88 small-molecule metabolites were identified based on retention time, accurate mass, and MS2 data, ensuring acceptable peak shapes and intensities across different software packages.

**4. The data standardization process of MetaboAnalyst 5.0**

The metabolites data were standardized using MetaboAnalyst 5.0. The following parameters were used in the standardized process of this study: (1) Missing value processing: elements with missing values > 80% were removed, and the missing values were replaced by 1/5 of minimum positive values of their corresponding variables. (2) Data filtering was carried out using median absolute deviation (median absolute deviation). (3) Data normalization (i.e., sample normalization, data transformation, and data scaling): Data normalization was performed by “normalization by the median”, “log transformation (base 10), and “auto-scaling (mean-centered and divided by the standard deviation of each variable)”. In the previous reports, MetaboAnalyst 5.0 were commonly used to standardize data processing and statistical analysis in metabolomics studies [7-10].

**5. The validation process of biomarkers diagnostic capabilities**

We recruited participants, including 25 GLL patients and 15 health controls (HCs), as an external validation set, and then used them to validate the potential biomarkers. Results from OPLS-DA analysis and heat-map showed that hexadecanamide could clearly differentiate between GLL patients and HCs. An ideal biomarker in the validation set should meet the following criteria: (1) revealed significant differences between the two groups under comparison (GLL vs. HCs), and (2) simultaneously maintained the same change trend as the discovery set for these comparisons. Furthermore, we generated AUC values and used them to evaluate the diagnostic ability of the biomarkers in GLL, and found that the 2 biomarkers had high AUC values, indicating that they have excellent diagnostic potential between GLL and HCs (Fig. 4d - Fig. 4f). Moreover, the results from a forward LR analysis revealed that the 2 biomarkers were reliable metabolites in the regression model. Furthermore, the biomarker was significantly elevated in GLL patients, relative to HCs, while ROC curves showed that hexadecanamide had high AUC values (> 0.9). In summary, these results indicated that hexadecanamide presented high diagnostic efficacy, and could be used to distinguish GLL patients from HCs. Consequently, hexadecanamide was defined as ideal biomarker panels for diagnosis of GLL. Finally, we analyzed the biological relevance between biomarker and BS to further ascertain which ones were ideal biomarkers for detecting GLL. Results showed that hexadecanamide positively correlated with the BS (Fig. 5). However, these results need to be validated at the molecular level in the further study.

**6. Metabolic pathways**

From Figure 6, size and color could present which metabolites mapped to which bubbles, and the small bubbles on the far left actually has “no hits” on it. Among 24 DEMs, arachidonic acid was mapped to α-linolenic acid and linoleic acid metabolism. However, hexadecanamide and propyl paraben were not mapped to α-linolenic acid and linoleic acid metabolism.

**References**

[1] Smith CA, Want EJ, O’Maille G, Abagyan R, Siuzdak G, XCMS: processing mass spectrometry data for metabolite profiling using nonlinear peak alignment, matching, and identification. Anal Chem. 2006; 78: 779-787.

[2] Gowda H, Ivanisevic J, Johnson CH, Kurczy ME, BentonHP, Rinehart D, et al. Interactive XCMS online: simplifying advanced metabolomic data processing and subsequent statistical analyses. Anal Chem. 2014; 86: 6931−6939.

[3] Myers OD, Sumner SJ, Li S, Barnes S, Du X, Detailed investigation and comparison of the XCMS and MZmine 2 chromatogram construction and chromatographic peak detection methods for preprocessing mass spectrometry metabolomics data. Anal Chem. 2017; 89: 8689−8695.

[4] Mahieu NG, Genenbacher JL, Patti GJ, A roadmap for the XCMS family of software solutions in metabolomics. Curr Opin Chem Biol. 2016; 30: 87−93.

[5] Shen X, Wang R, Xiong X, Yin Y, Cai Y, Ma Z, et al. Metabolic reaction network-based recursive metabolite annotation for untargeted metabolomics. Nat Commun. 2019; 10: 1516.

[6] Yin Y, Wang R, Cai Y, Wang Z, Zhu Z, DecoMetDIA: deconvolution of multiplexed MS/MS spectra for metabolite identification in SWATH-MS-based untargeted metabolomics. Anal Chem. 2019; 91: 11897–11904.

[7] Hou X, Wang Y, Ke C, Pan C, Metabolomics facilitates the discovery of metabolic profiles and pathways for myopia: A systematic review. Eye. 2022; 37: 670-677.

[8] Wang Y, Guo F, Hao D, Guo Y, Xu T, Shen Q, et al. Nontargeted serum metabolomics analysis and potential biomarkers for systemic lupus erythematosus. Microchem J. 2021; 170: 106677.

[9] Salamoun YM, Polireddy K, Kyoung Cho Y, Funk RS, Metabolomic profiling of red blood cells to identify molecular markers of methotrexate response in the collagen induced arthritis mouse model. Future Pharmacol. 2022; 2 (4): 625-641.

[10] Xia J, Wishart DS, Web-based inference of biological patterns, functions and pathways from metabolomic data using MetaboAnalyst. Nat Protoc. 2011; 6: 743–760.
