# Supplementary figures and images for: Clinical metabolomics reveals potential diagnostic biomarkers in serum samples from patients with generalized ligamentous laxity
Source: Front Mol Biosci. 2025 May 30;12:1554936. doi: 10.3389/fmolb.2025.1554936 (PMC12162281; doi:10.3389/fmolb.2025.1554936)

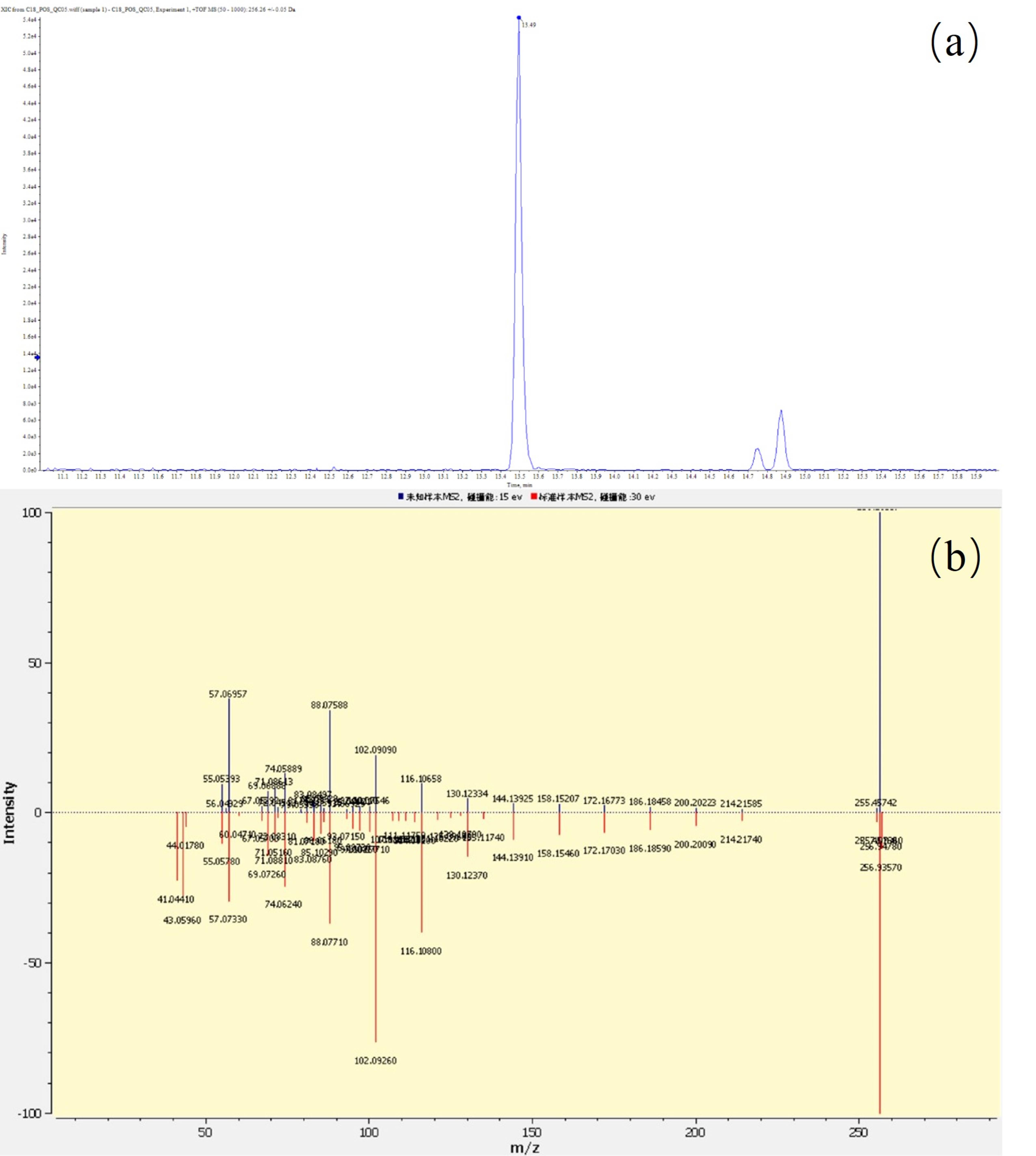

Supplement: Supplementary file 2 [file Image1.jpeg]

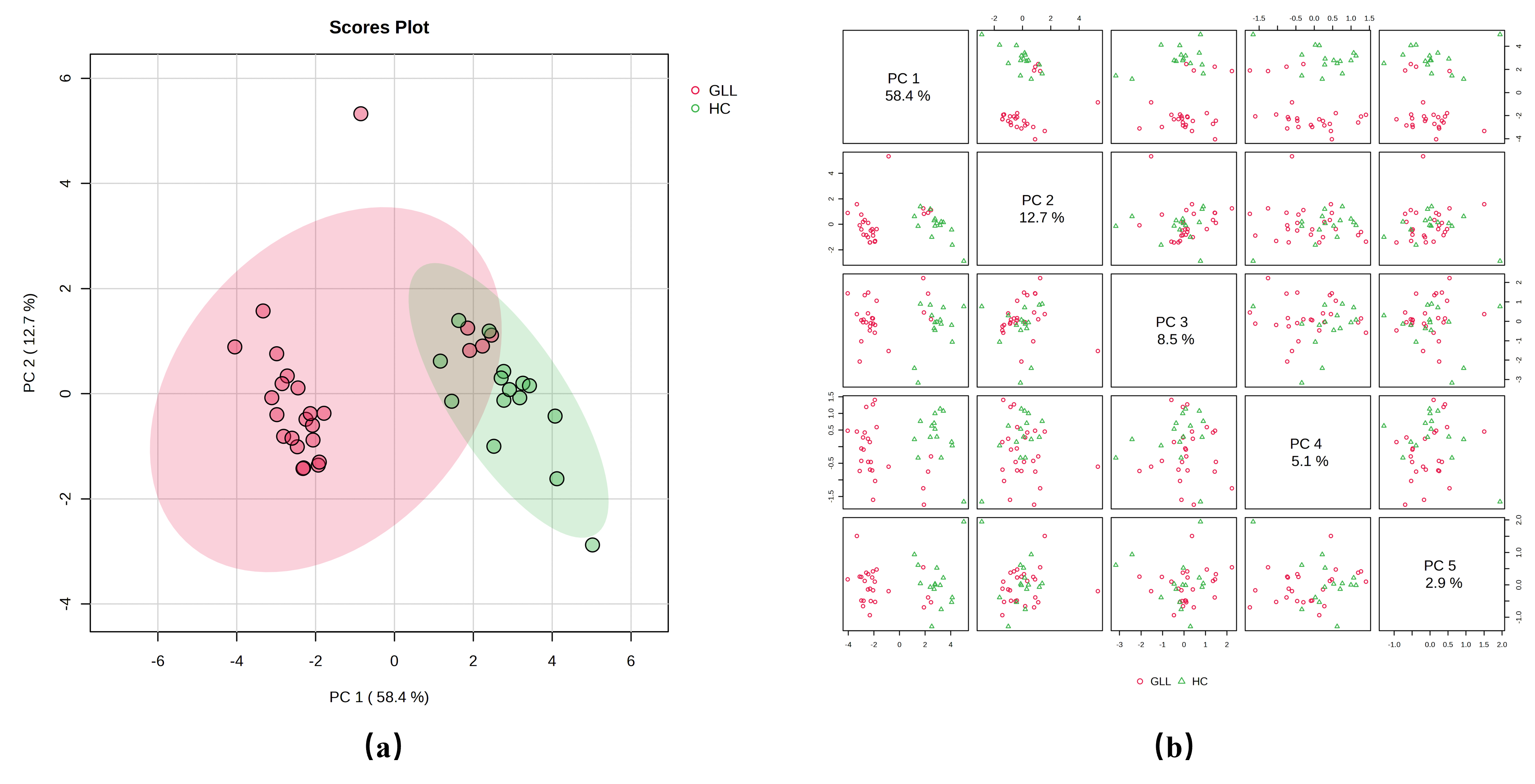

Supplement: Supplementary file 3 [file Image2.jpeg]
