# Supplementary material for: Clinical metabolomics reveals potential diagnostic biomarkers in serum samples from patients with generalized ligamentous laxity
Source: Front Mol Biosci. 2025 May 30;12:1554936. doi: 10.3389/fmolb.2025.1554936 (PMC12162281; doi:10.3389/fmolb.2025.1554936)
Supplement: Supplementary file 4 [file DataSheet1.docx]

**Table S1** The important identified information of 38 DEMs

| **DEMs** | **Formula** | **CAS Number** | **tR(s)** | **m/z (Da)** |
| --- | --- | --- | --- | --- |
| 1-Oleoyl-Sn-Glycero-3-Phosphocholine | C_26_H_52_NO_7_P | 19420-56-5 | 692.6515 | 522.3565 |
| 1-Palmitoyl-Sn-Glycero-3-Phosphocholine | C_24_H_50_NO_7_P | 17364-16-8 | 673.124 | 496.3409 |
| 2-Oxovaleric acid | C_5_H_8_O_3_ | 1821-02-9 | 172.644 | 115.0395 |
| 3-Methyl-2-Oxovalerate | C_6_H_10_O_3_ | 1460-34-0 | 69.4565 | 129.055 |
| 4-Hydroxybenzoic acid | C_7_H_6_O3 | 99-96-7 | 522.506 | 139.0389 |
| 7-Ethoxy-4-methyl-2h-chromen-2-one | C_12_H_12_O_3_ | 1987/5/8 | 732.127 | 205.0862 |
| 7-Ketodeoxycholic acid | C_24_H_38_O_5_ | 911-40-0 | 75.2275 | 405.2631 |
| All-cis-4,7,10,13,16-docosapentaenoic acid | C_22_H_34_O_2_ | 25182-74-5 | 77.367 | 329.2474 |
| Arachidonic acid | C_20_H_32_O_2_ | 506-32-1 | 77.332 | 303.2322 |
| Cholesteryl laurate | C_39_H_68_O_2_ | 1908/11/8 | 123.661 | 283.2637 |
| Cis-8,11,14-Eicosatrienoic acid | C_20_H_34_O_2_ | 1783-84-2 | 77.5645 | 305.2473 |
| Citric acid | C_6_H_8_O_7_ | 77-92-9 | 723.009 | 191.0191 |
| Crystal Violet | C_25_H_29_N_3_ | 548-62-9 | 574.848 | 372.2436 |

**Table S2** 38 DEMs with significant changes screened from the discovery Set

| **DEMs** | **^a^VIP** | **^b^P.value** | **^c^FDR** | **^d^FC** | **log2(FC)** |
| --- | --- | --- | --- | --- | --- |
| 1-Oleoyl-Sn-Glycero-3-Phosphocholine | 1.099 | <0.001 | <0.001 | 1.662 | 0.733 |
| 1-Palmitoyl-Sn-Glycero-3-Phosphocholine | 1.202 | <0.001 | <0.001 | 1.854 | 0.890 |
| 2-Oxovaleric acid | 1.341 | <0.001 | <0.001 | 2.552 | 1.351 |
| 3-Methyl-2-Oxovalerate | 2.093 | <0.001 | <0.001 | 27.322 | 4.772 |
| 4-Hydroxybenzoic acid | 1.412 | <0.001 | <0.001 | 0.438 | -1.192 |
| 7-Ethoxy-4-methyl-2h-chromen-2-one | 1.735 | <0.001 | <0.001 | 3.429 | 1.778 |
| 7-Ketodeoxycholic acid | 1.999 | <0.001 | <0.001 | 0.122 | -3.033 |
| All-cis-4,7,10,13,16-docosapentaenoic acid | 1.586 | <0.001 | <0.001 | 0.349 | -1.520 |
| Arachidonic acid | 1.503 | <0.001 | <0.001 | 0.288 | -1.798 |
| Cholesteryl laurate | 1.652 | <0.001 | <0.001 | 0.266 | -1.913 |
| Cis-8,11,14-Eicosatrienoic acid | 1.479 | <0.001 | <0.001 | 0.425 | -1.236 |
| Citric acid | 1.730 | <0.001 | <0.001 | 21.410 | 4.420 |
| Crystal Violet | 1.208 | <0.001 | <0.001 | 3.426 | 1.777 |
| delta-Valerolactam | 1.345 | <0.001 | <0.001 | 0.442 | -1.178 |
| Dl-Lactic acid | 1.271 | <0.001 | <0.001 | 2.133 | 1.093 |
| D-Norvaline | 1.166 | <0.001 | <0.001 | 0.438 | -1.192 |
| Docosahexaenoic acid | 1.449 | <0.001 | <0.001 | 0.407 | -1.296 |
| Dulcitol | 1.645 | <0.001 | <0.001 | 5.092 | 2.348 |
| gamma-Linolenic acid | 1.041 | 0.001 | 0.002 | 0.604 | -0.728 |
| Glucoheptonic acid | 1.204 | <0.001 | <0.001 | 2.089 | 1.063 |
| Gluconate | 1.477 | <0.001 | <0.001 | 6.404 | 2.679 |
| Hexadecanamide | 1.571 | <0.001 | <0.001 | 7.581 | 2.922 |
| Hypoxanthine | 1.086 | <0.001 | <0.001 | 0.425 | -1.235 |
| Inosine | 1.061 | <0.001 | <0.001 | 1.138 | 0.186 |
| L-Carnitine | 1.274 | <0.001 | <0.001 | 2.038 | 1.027 |
| L-Cysteinesulfinic acid | 1.055 | <0.001 | <0.001 | 1.645 | 0.718 |
| Myoinositol | 1.115 | <0.001 | <0.001 | 2.212 | 1.145 |
| N-Acetylaspartate | 1.572 | <0.001 | <0.001 | 16.124 | 0.169 |
| Oleamide | 1.633 | <0.001 | <0.001 | 7.546 | 2.916 |
| Oleoyl-L-alpha-lysophosphatidic acid | 1.194 | <0.001 | <0.001 | 0.466 | -1.101 |
| Oxypurinol | 1.083 | <0.001 | <0.001 | 0.379 | -1.399 |
| Palmitic acid | 1.064 | <0.001 | <0.001 | 0.621 | -0.688 |
| Phe-Phe | 1.750 | <0.001 | <0.001 | 0.127 | -2.978 |
| Phloroglucinol carboxylic acid | 1.039 | <0.001 | <0.001 | 1.704 | 0.769 |
| Phosphoric acid | 1.312 | <0.001 | <0.001 | 0.420 | -1.252 |
| Propylparaben | 1.606 | <0.001 | <0.001 | 0.230 | -2.119 |
| Purine | 1.202 | <0.001 | <0.001 | 2.005 | 1.003 |
| trans-Vaccenic acid | 1.181 | <0.001 | <0.001 | 0.527 | -0.923 |

^a^The VIP value was obtained from the OPLS-DA model with a threshold of 1.0. ^b^P values were obtained from one-way ANOVA. ^c^The value of FDR was obtained from the adjusted P value calculated using MetaboAnalyst 5.0 software. ^d^The value of FC was obtained by comparing metabolites between patients with GLL with HCs.

**Table S3** The 119 metabolites that can be qualitatively identified

| **DEMs** | **Formula** | **CAS**  **Number** | **tR(s)** | **m/z (Da)** |
| --- | --- | --- | --- | --- |
| Ciprofloxacin | C17H18FN3O3 | 85721-33-1 | 721.922 | 661.2528 |
| trans-9-Octadecenoic acid | C18H34O2 | 112-79-8 | 77.405 | 563.5018 |
| Oleoyl-L-α-lysophosphatidic acid | C21H41O7P | 22556-62-3 | 337.8305 | 457.2342 |
| Glycoursodeoxycholic acid | C26H43NO5 | 64480-66-6 | 295.162 | 448.3047 |
| 25-Hydroxycholesterol | C27H46O2 | 2140-46-7 | 82.586 | 447.3459 |
| Flurandrenolide | C24H33FO6 | 1524-88-5 | 63.918 | 417.2095 |
| Pentadecafluorooctanoic acid | C8HF15O2 | 335-67-1 | 563.571 | 412.9639 |
| D(+)-Tryptophan | C11H12N2O2 | 153-94-6 | 493.452 | 407.1701 |
| 7-Ketodeoxycholic acid | C24H38O5 | 911-40-0 | 75.2275 | 405.2631 |
| Methyl prednisolone acetate | C24H32O6 | 53-36-1 | 69.2355 | 397.2037 |
| 4-Pyridoxic acid | C8H9NO4 | 82-82-6 | 376.302 | 365.1021 |
| D-Ribulose 1,5-bisphosphate | C5H12O11P2 | 14689-84-0 | 563.556 | 330.9621 |
| All-cis-4,7,10,13,16-docosapentaenoic acid | C22H34O2 | 25182-74-5 | 77.367 | 329.2474 |
| Docosahexaenoic acid | C22H32O2 | 6217-54-5 | 75.504 | 327.2306 |
| 13-Cis-Acitretin | C21H26O3 | 69427-46-9 | 62.6175 | 325.1834 |
| Phe-Phe | C18H20N2O3 | 2577-40-4 | 385.0645 | 311.1394 |
| Cis-8,11,14-Eicosatrienoic acid | C20H34O2 | 1783-84-2 | 77.5645 | 305.2473 |
| Arachidonic acid | C20H32O2 | 506-32-1 | 77.332 | 303.2322 |
| D-Norvaline | C5H11NO2 | 2013-12-9 | 56.859 | 293.1786 |
| Ethylenediaminetetraacetic acid | C10H16N2O8 | 60-00-4 | 886.034 | 291.0824 |
| Ethylenediaminetetraacetic acid | C10H16N2O8 | 60-00-4 | 961.4915 | 291.0825 |
| Ethylenediaminetetraacetic acid | C10H16N2O8 | 60-00-4 | 922.979 | 291.0826 |
| Stearic acid | C18H36O2 | 57-11-4 | 77.408 | 283.2635 |
| Cholesteryl laurate | C39H68O2 | 1908-11-8 | 123.661 | 283.2637 |
| Cholesteryl laurate | C39H68O2 | 1908-11-8 | 223.231 | 283.2635 |
| trans-Vaccenic acid | C18H34O2 | 693-72-1 | 77.2165 | 281.2481 |
| O-Phosphorylethanolamine | C2H8NO4P | 1071-23-4 | 310.5695 | 281.0364 |
| β-Linoleic acid | C18H32O2 | 60-33-3 | 77.752 | 279.2326 |
| Uridine | C9H12N2O6 | 58-96-8 | 307.659 | 279.038 |
| γ-Linolenic acid | C18H30O2 | 506-26-3 | 78.1595 | 277.2168 |
| Inosine | C10H12N4O5 | 58-63-9 | 399.318 | 267.0729 |
| Phenylacetyl-L-glutamine | C13H16N2O4 | 28047-15-6 | 348.965 | 263.1031 |
| Palmitic acid | C16H32O2 | 57-10-3 | 222.551 | 255.2324 |
| Palmitic acid | C16H32O2 | 57-10-3 | 78.127 | 255.2324 |
| Phenyl-β-D-glucopyranoside | C12H16O6 | 1464-44-4 | 196.398 | 255.0867 |
| Palmitoleic acid | C16H30O2 | 373-49-9 | 78.6295 | 253.2168 |
| Uridine | C9H12N2O6 | 58-96-8 | 309.295 | 243.0616 |
| Uridine | C9H12N2O6 | 58-96-8 | 457.64 | 243.0617 |
| Cytidine | C9H13N3O5 | 65-46-3 | 735.565 | 242.0791 |
| Glucoheptonic acid | C7H14O8 | 23351-51-1 | 573.765 | 225.0608 |
| Glucoheptonic acid | C7H14O8 | 23351-51-1 | 747.756 | 225.061 |
| Glucoheptonic acid | C7H14O8 | 23351-51-1 | 544.4055 | 225.0609 |
| Sulfosalicylic acid | C7H6O6S | 97-05-2 | 79.592 | 216.9811 |
| 1,3-Dimethyluric acid | C7H8N4O3 | 944-73-0 | 543.291 | 217.0292 |
| D-Galactose | C6H12O6 | 59-23-4 | 455.621 | 215.0322 |
| D-Galactose | C6H12O6 | 59-23-4 | 488.0975 | 215.032 |
| D-Galactose | C6H12O6 | 59-23-4 | 543.2185 | 215.0322 |
| Indoxyl sulfate | C8H7NO4S | 2642-37-7 | 64.3725 | 212.0018 |
| 3,5-Dinitrobenzoic acid | C7H4N2O6 | 99-34-3 | 762.143 | 211.0025 |
| L-Tryptophan | C11H12N2O2 | 73-22-3 | 493.233 | 203.082 |
| Allo-inositol | C6H12O6 | 643-10-7 | 372.363 | 201.0342 |
| Gluconate | C6H12O7 | 526-95-4 | 722.408 | 195.0503 |
| 3-Hydroxyhippuric acid | C9H9NO4 | 1637-75-8 | 106.163 | 194.0489 |
| Citric acid | C6H8O7 | 77-92-9 | 866.9015 | 191.0189 |
| Citric acid | C6H8O7 | 77-92-9 | 812.387 | 191.0191 |
| Citric acid | C6H8O7 | 77-92-9 | 759.3795 | 191.0191 |
| Citric acid | C6H8O7 | 77-92-9 | 723.009 | 191.0191 |
| 1-Hydroxy-2-Naphthoic Acid | C11H8O3 | 86-48-6 | 239.766 | 187.0415 |
| Dl-3,4-Dihydroxymandelic acid | C8H8O5 | 775-01-9 | 160.4795 | 183.0331 |
| Dulcitol | C6H14O6 | 608-66-2 | 535.145 | 181.0711 |
| L-Methionine sulfone | C5H11NO4S | 7314-32-1 | 137.393 | 180.0334 |
| Dl-Tyrosine | C9H11NO3 | 556-03-6 | 576.98 | 180.0658 |
| Myoinositol | C6H12O6 | 87-89-8 | 504.6 | 179.0555 |
| Propylparaben | C10H12O3 | 94-13-3 | 79.646 | 179.0707 |
| Myoinositol | C6H12O6 | 87-89-8 | 573.384 | 179.0555 |
| Myoinositol | C6H12O6 | 87-89-8 | 544.785 | 179.0555 |
| Hippuric acid | C9H9NO3 | 495-69-2 | 225.849 | 178.0505 |
| N-Acetylaspartate | C6H9NO5 | 997-55-7 | 826.796 | 174.0403 |
| D(-)-Arginine | C6H14N4O2 | 157-06-2 | 876.555 | 173.104 |
| D(-)-Arginine | C6H14N4O2 | 157-06-2 | 903.759 | 173.1039 |
| Nα-Acetyl-L-ornithine | C7H14N2O3 | 6205-08-9 | 670.184 | 173.0926 |
| 3-Nitrobenzoic acid | C7H5NO4 | 121-92-6 | 146.4965 | 166.0175 |
| L-Phenylalanine | C9H11NO2 | 63-91-2 | 485.086 | 164.0712 |
| 1,6-Anhydro-β-D-Glucopyranose | C6H10O5 | 498-07-7 | 544.99 | 161.0449 |
| Allantoin | C4H6N4O3 | 97-59-6 | 354.166 | 157.0361 |
| D-Histidine | C6H9N3O2 | 351-50-8 | 895.155 | 154.0617 |
| L-Histidine | C6H9N3O2 | 71-00-1 | 826.417 | 154.0617 |
| L-Cysteinesulfinic acid | C3H7NO4S | 1115-65-7 | 164.133 | 152.0017 |
| Phloroglucinol carboxylic acid | C7H6O5 | 83-30-7 | 79.087 | 151.0066 |
| Oxypurinol | C5H4N4O2 | 2465-59-0 | 356.8485 | 151.0255 |
| D-Methionine | C5H11NO2S | 348-67-4 | 531.503 | 148.0432 |
| trans-Cinnamic acid | C9H8O2 | 140-10-3 | 485.2365 | 147.0445 |
| Indole-3-carbinol | C9H9NO | 700-06-1 | 719.919 | 146.0645 |
| L-Glutamic acid | C5H9NO4 | 56-86-0 | 759.357 | 146.0454 |
| L-Glutamine | C5H10N2O3 | 56-85-9 | 719.5685 | 145.0613 |
| Adipamide | C6H12N2O2 | 628-94-4 | 826.513 | 143.0821 |
| Hypoxanthine | C5H4N4O | 68-94-0 | 642.259 | 135.0293 |
| Hypoxanthine | C5H4N4O | 68-94-0 | 311.981 | 135.0306 |
| Dl-Aspartic acid | C4H7NO4 | 617-45-8 | 811.2975 | 132.0297 |
| L(+)-Ornithine | C5H12N2O2 | 70-26-8 | 877.2015 | 131.082 |
| L(+)-Ornithine | C5H12N2O2 | 70-26-8 | 919.807 | 131.0821 |
| L(+)-Ornithine | C5H12N2O2 | 70-26-8 | 757.8795 | 131.0819 |
| L-Asparagine | C4H8N2O3 | 70-47-3 | 734.665 | 131.0455 |
| (2R,3R)-2-Amino-3-methylpentanoic acid | C6H13NO2 | 319-78-8 | 508.436 | 130.0868 |
| L-Hydroxyproline | C5H9NO3 | 51-35-4 | 649.5305 | 130.0503 |
| D-Leucine | C6H13NO2 | 328-38-1 | 489.055 | 130.0868 |
| 3-Methyl-2-Oxovalerate | C6H10O3 | 1460-34-0 | 69.4565 | 129.055 |
| 2-Ketohexanoic acid /2-oxohexanoic acid | C6H10O3 | 2492-75-3 | 158.762 | 129.0551 |
| 2-Ketohexanoic acid /2-oxohexanoic acid | C6H10O3 | 2492-75-3 | 107.292 | 129.0552 |
| Dl-Glutamic acid | C5H9NO4 | 617-65-2 | 759.6 | 128.0347 |
| L-Glutamic acid | C5H9NO4 | 56-86-0 | 719.8205 | 128.0346 |
| Dl-Glutamic acid | C5H9NO4 | 617-65-2 | 578.076 | 128.0346 |
| 4-Chlorophenol | C6H5ClO | 106-48-9 | 563.856 | 127.0005 |
| L-Glutamine | C5H10N2O3 | 56-85-9 | 719.764 | 127.0505 |
| Purine | C5H4N4 | 120-73-0 | 544.148 | 119.0342 |
| Dl-Threonine | C4H9NO3 | 72-19-5 | 671.186 | 118.0502 |
| 2-Hydroxy-3-methylbutyric acid | C5H10O3 | 4026-18-0 | 174.184 | 117.0551 |
| D-Norvaline | C5H11NO2 | 2013-12-9 | 559.457 | 116.071 |
| 2-Oxovaleric acid | C5H8O3 | 1821-02-9 | 172.644 | 115.0395 |
| 2-Oxovaleric acid | C5H8O3 | 1821-02-9 | 73.44 | 115.0395 |
| m-Cresol | C7H8O | 108-39-4 | 56.231 | 107.0496 |
| α-Hydroxyisobutyric acid | C4H8O3 | 594-61-6 | 198.246 | 103.0396 |
| Phosphoric acid | H3O4P | 7664-38-2 | 73.658 | 96.95963 |
| Phosphoric acid | H3O4P | 7664-38-2 | 937.4885 | 96.95956 |
| Phosphoric acid | H3O4P | 7664-38-2 | 901.812 | 96.95935 |
| Phenol | C6H6O | 108-95-2 | 56.64 | 93.03407 |
| L(+)-Lactic acid | C3H6O3 | 79-33-4 | 242.1485 | 89.02388 |
| Dl-Lactic acid | C3H6O3 | 50-21-5 | 544.414 | 89.02379 |
| (R)-(+)-Lactamide | C3H7NO2 | 598-81-2 | 654.0385 | 88.03983 |
